# Impact of Maternal Country of Birth on Type-1-Diabetes Therapy and Outcome in 27,643 Children and Adolescents from the DPV Registry

**DOI:** 10.1371/journal.pone.0135178

**Published:** 2015-08-21

**Authors:** Nicole Scheuing, Susanna Wiegand, Christina Bächle, Elke Fröhlich-Reiterer, Eva Hahn, Andrea Icks, Karl-Heinz Ludwig, Kirsten Mönkemöller, Oliver Razum, Joachim Rosenbauer, Reinhard W. Holl, DPV initiative

**Affiliations:** 1 Institute of Epidemiology and Medical Biometry, Central Institute for Biomedical Technology, University of Ulm, German Center for Diabetes Research (DZD), Ulm, Germany; 2 Department of Pediatric Endocrinology and Diabetology, Charité Universitätsmedizin, Berlin, Germany; 3 Institute for Biometrics and Epidemiology, German Diabetes Center, German Center for Diabetes Research (DZD), Düsseldorf, Germany; 4 Department of Pediatrics, Medical University of Graz, Graz, Austria; 5 Department of Pediatrics and Adolescent Medicine, Protestant Hospital Oberhausen, Oberhausen, Germany; 6 Department of Public Health, Heinrich-Heine University Düsseldorf, Düsseldorf, Germany; 7 Clinic for Children and Adolescent Medicine, Mutterhaus der Borromäerinnen, Trier, Germany; 8 Children’s Hospital, Hospitals of the City of Cologne, Cologne, Germany; 9 Department of Epidemiology & International Public Health, School of Public Health, Bielefeld University, Bielefeld, Germany; National Institute of Health, ITALY

## Abstract

**Objective:**

To study the impact of maternal country of birth on type-1-diabetes (T1D) therapy and outcome.

**Study Design and Methods:**

27,643 T1D patients aged ≤20 years with documented maternal country of birth from the multicenter German/Austrian diabetes patient registry (DPV) were analyzed. Patients were categorized based on their mother’s origin: Germany/Austria (reference), Turkey, Southern Europe, and Eastern Europe. To compare BMI standard deviation score (BMI-SDS), diabetes therapy and outcome between groups, multivariable regression was applied with adjustments for age, sex and duration of diabetes. Based on observed marginal frequencies, adjusted estimates were calculated. Linear regression was used for continuous data, logistic regression for binary data and Poisson regression for count data. All statistical analyses were performed using SAS 9.4. Significance was set at a two-tailed p<0.05.

**Results:**

83.3% of patients were offspring of native mothers. A Turkish, Southern or Eastern European background was documented in 2.4%, 1.7% and 4.3% of individuals. After demographic adjustment, patients with migration background had a higher mean BMI-SDS (Turkey, Southern Europe or Eastern Europe vs. Germany/Austria: 0.58±0.03, 0.40±0.04, or 0.37±0.02 vs. 0.31±0.01; ±SE) and a lower use of insulin pumps (26.8%, 27.9%, or 32.6% vs. 37.9%) compared to offspring of native mothers. Mean HbA1c was worst in individuals of Turkish mothers (Turkey vs. Germany/Austria: 69.7±0.7 vs. 66.6±0.1 mmol/mol; ±SE). Patients of Eastern European descent had an increased rate of severe hypoglycemia (22.09±0.13 vs. 16.13±0.02 events per 100 patient-years) and ketoacidosis was more prevalent in offspring of Turkish or Southern European mothers (7.50±0.10, or 7.13±0.11 vs. 6.54±0.02 events per 100 patient-years). Patients of Turkish descent were more often hospitalized (57.2±2.7 vs. 48.5±0.4 per 100 patient-years). All differences were significant.

**Conclusion:**

The differences in diabetes therapy and outcome among patients with distinct migration background suggest that specific challenges have to be considered in clinical care.

## Introduction

Type-1-diabetes (T1D) is the most common chronic metabolic disease in childhood and adolescence. In Germany, approximately 30,000 children and adolescents are affected [1]. A very large number of studies revealed an association between migration and poor health status in T1D [2–6]. However, health status varies widely among ethnicities and depends on the country of origin [7]. In Germany, about one quarter of children and adolescents under the age of 18 years has a migration background and people of Turkish descent are the largest migrant population [7]. Nowadays, the majority (80%) of migrant children and adolescents is born in Germany and are second- or third-generation descendants of immigrants [7].

With rising cultural diversity, challenges in daily life and health care occur [8]. It is known that with longer duration of stay immigrants tend to adopt living habits, health concepts and health-related behavior of the new country [9,10]. However, their health status may worsen due to a lower socio-economic status or migration stress [9,10]. In people with migration background, cultural or religious differences, different health belief or self-care behavior and a lower adherence to treatment regimens compared to the native population were also described and may additionally contribute to a worse health outcome [3,4,11]. Even though a recent Canadian study found no increased rate of diabetes complications in immigrants with language barriers [12], communication problems have been also reported to result in difficulties during routine care [4]. Lower health literacy skills, that mean a lower ability to gather, understand and apply health information in order to make adequate judgements and decisions in healthcare and to adhere to treatment recommendations [13], might be an additional contributor to a worse health status in immigrants compared to the native population [14]. Furthermore, nutritional and lifestyle habits differ between ethnicities and cultural knowledge is often lacking in health care professionals [8]. As known for obesity and T1D incidence [15,16], a genetic component has also been reported to play a role in glycemic control and the occurrence of diabetes-related complications, mainly retinopathy and nephropathy [16,17]. Thereby, differences in health outcome can also partially be attributed to specific genomic loci that influence disease susceptibility and differ between certain population groups [16,17].

There are several studies reporting a poor glycemic control, a higher rate of hospitalization, hypoglycemia or ketoacidosis, and finally an increased risk for late diabetes-related complications and mortality in patients with migrant background [3–6,18]. By contrast, in the Hvidoere study [4], patients with language difficulties had no increased frequency of diabetic ketoacidosis or hypoglycemia. Due to the inconsistent definitions and concepts of ethnicity or migrant background between populations and studies [19], a direct comparison is often difficult and a transfer from findings in foreign countries to the own country is not possible. In particular for children of immigrants, a higher hemoglobin A1c (HbA1c) and a lower use of insulin pumps compared to children of native mothers have been reported [20]. As lack of parental support has been associated with higher HbA1c [21], potential cultural discrepancies for parental involvement in diabetes management may also contribute to differences in children’s health outcome. Differences in usage of and access to pump therapy or high-quality medical care were discussed as potential reasons for the lower use of insulin pumps in migrant groups [22]. In children, daily diabetes management is usually supervised by mothers rather than fathers, and mostly mothers are the ones caring for the child [23].

The primary aim of this study was to investigate the influence of maternal country of birth on T1D therapy and outcome in 27,643 patients aged 20 years or younger. Contrary to many previous studies, this research differentiates between regions of origin, as individuals with migration background are not a homogenous group.

## Methods

### Ethics statement

The DPV initiative has been approved by the Ethical Committee of the Medical Faculty of the University of Ulm, Germany and the anonymized data collection by the local review board of each participating center.

### Patients and data collection

Data from the multicenter, standardized, prospective diabetes patient registry, DPV, was used for the present study (www.d-p-v.eu). Over a period of nearly 20 years, specialized diabetes care centers all over Germany and Austria have been documenting patient characteristics and diabetes-specific data regularly in an electronic health record system. Semi-annually, the locally documented data is transferred in anonymous form to Ulm University, Germany. After plausibility checks and corrections, the data is aggregated into a cumulative database, the DPV registry. It covers an estimated proportion of >80% of all pediatric patients diagnosed with diabetes in Germany and Austria [24].

**Fig 1:**
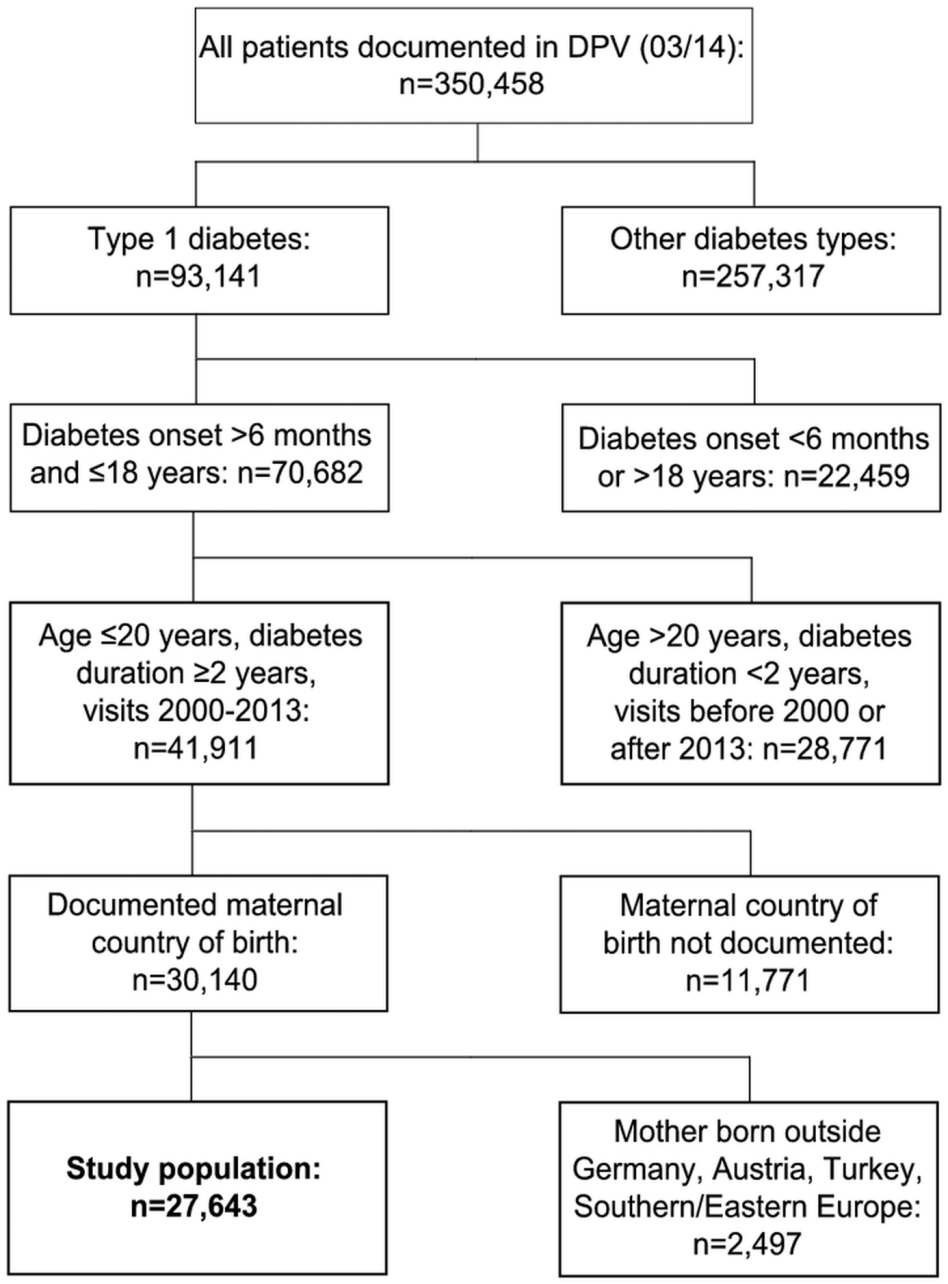
Selection of study population.

For the present study, ≤20 year-old patients with T1D onset between >6 months and ≤18 years and at least 2 years of diabetes were included (Fig 1). Further inclusion criteria were: documented visits between 2000 and 2013, and information on maternal country of birth. Patients with mothers born outside Germany/Austria, Turkey, Southern or Eastern Europe were excluded due to the small number of cases per country. Finally, 27,643 T1D patients were eligible for the study (Fig 1). The most recent treatment year of each patient was analyzed. In case of multiple datasets per patient, quantitative parameters were aggregated as median and count data as cumulative sums.

### Definition of migration status

Migration status was defined based on maternal country of birth. The study population was categorized into four groups according to patient’s maternal origin: i) Germany/Austria (native mothers; reference), ii) Turkey, iii) Southern Europe, and iv) Eastern Europe. The definitions for Southern and Eastern Europe were primarily based on geographic criteria [25], modified by economic and cultural aspects. Southern Europe comprised Albania, Andorra, Gibraltar, Greece, Vatican City State, Italy, Malta, Portugal, San Marino, Spain, former Yugoslavia, and excluded Turkey. Bulgaria, Czech Republic, Hungary, Poland, Romania, Slovakia and the countries of the former Soviet Union formed Eastern Europe.

### Anthropometry

Body mass index standard deviation score (BMI-SDS) was computed by using national reference data from the German Health Interview and Examination Survey for Children and Adolescents (KiGGS, Robert Koch-Institute, Berlin, Germany) [26]. For patients aged >18 years, data was extrapolated by using L-, M- and S-values of 18 year-old people. In the KiGGS study, 17% of all participants had a migration background [26].

### Glycemic control

Glycemic control was assessed by median HbA1c. The multiple of the mean method was applied to mathematically harmonize HbA1c values to the Diabetes Control and Complications Trial reference range (20.7–42.6 mmol/mol; 4.05–6.05%) [6,27].

### Diabetes therapy

Insulin treatment was categorized as conventional injection therapy using syringes/pens (1–8 injection time-points/day) and insulin pump therapy. Daily insulin dosage per kilogram body weight was calculated and the number of injection time-points per day was analyzed by using the definition that insulin pump therapy equals 9 injection time-points per day. Daily frequency of self-monitoring of blood glucose (SMBG) was evaluated.

### Acute diabetes-related complications

If patients required assistance of another person during an episode of hypoglycemia to administer carbohydrates or glucagon, the event was specified as severe hypoglycemia. Diabetic ketoacidosis (DKA) was defined as either a blood pH value <7.3, or as a clinician-based diagnosis of diabetic ketoacidosis associated with hospitalization.

### Chronic diabetes-related complications

Hypertension was classified as an elevated median systolic or diastolic blood pressure ≥95th percentile for age, sex and height and/or the use of anti-hypertensive medication. Reference values for normative blood pressure were retrieved from the KiGGS study [28]. In patients aged >18 years, values were extrapolated. Dyslipidemia was classified as the use of lipid-lowering medication and/or at least one lipid parameter on average above/below the following thresholds: total cholesterol >5.2 mmol/l, triglycerides >1.7 mmol/l, LDL >3.4 mmol/l, HDL <0.9 mmol/l [29]. Microalbuminuria was assessed by urinary albumin excretion. In line with the American Diabetes Association (ADA) guidelines, a value of ≥30 mg/24 h (≥20 μg/min on a timed sample or ≥30mg/g creatinine on a random collection) in at least two of three tests was defined as microalbuminuria [30]. As clinical manifestation of micro- and macrovascular complications of diabetes are uncommon in childhood and adolescence [31], further chronic diabetes-related complications like retinopathy, stroke, myocardial infarction or diabetic foot syndrome could not be analyzed in our group of pediatric patients.

### Patient care

To specify patient care, the rate of hospitalization and the duration of hospital stay were studied. Moreover, the number of outpatient visits was analyzed.

### Statistical analysis

Results of descriptive statistics are displayed as median with quartiles or as proportion. As non-normally distributed data was assumed, Kruskal-Wallis test was used to compare continuous parameters and χ^2^-test was applied for dichotomous variables.

Due to differences in demographics between patients with migration background and native patients, multivariable regression models were created to compare BMI-SDS, diabetes therapy and outcome. Adjustments were made for the following demographics: age, sex and duration of diabetes. Based on observed marginal frequencies, adjusted estimates were calculated. Linear regression was applied for continuous data and logistic regression for binary data. Count data was compared by Poisson regression. As estimation technique, residual pseudo-likelihood with a subject-specific expansion (rspl) was used in linear regression and maximum pseudo-likelihood with marginal expansion (mmpl) in logistic or Poisson regression. To investigate whether paternal influence on diabetes management is similar to that of the mother, the analyses were repeated after categorizing patients by their father’s origin of birth (Germany/Austria: n = 24,948, Turkey: n = 788, Southern Europe: n = 599, Eastern Europe: n = 1,122, unknown: n = 186).

SAS version 9.4 (Statistical Analysis Software, SAS Institute Inc., Cary, NC, USA) was used for statistical analysis. Significance was set at a two-tailed p<0.05.

## Results

### Description of study population

Median age of the 27,643 patients (48.1% girls) eligible for the study was 15.7 [12.4; 17.6] years and median duration of diabetes amounted to 6.3 [4.0; 9.4] years. 83.3% (n = 25,107) of patients studied had a mother born in Germany or Austria. A Turkish or Southern European, or Eastern European background was documented in 2.4% (n = 736), 1.7% (n = 517) and 4.3% (n = 1,283) of individuals. In group-specific analysis, median age and duration of diabetes was higher in patients of native mothers compared to offspring of non-native mothers (Germany/Austria vs. Turkey, Southern or Eastern Europe; age in years: 15.7 [12.5; 17.5] vs. 14.8 [12.1; 17.4], 14.4 [10.9; 17.0], or 14.3 [11.0; 17.1], each p<0.001; duration of diabetes in years: 6.4 [4.0; 9.4] vs. 5.8 [3.7; 8.8], 5.9 [3.9; 9.1], or 5.6 [3.7; 8.3], each p<0.001). Proportion of girls did not differ significantly between groups (47.8 vs. 52.3, 49.3, or 49.4%).

### Anthropometry

**Fig 2:**
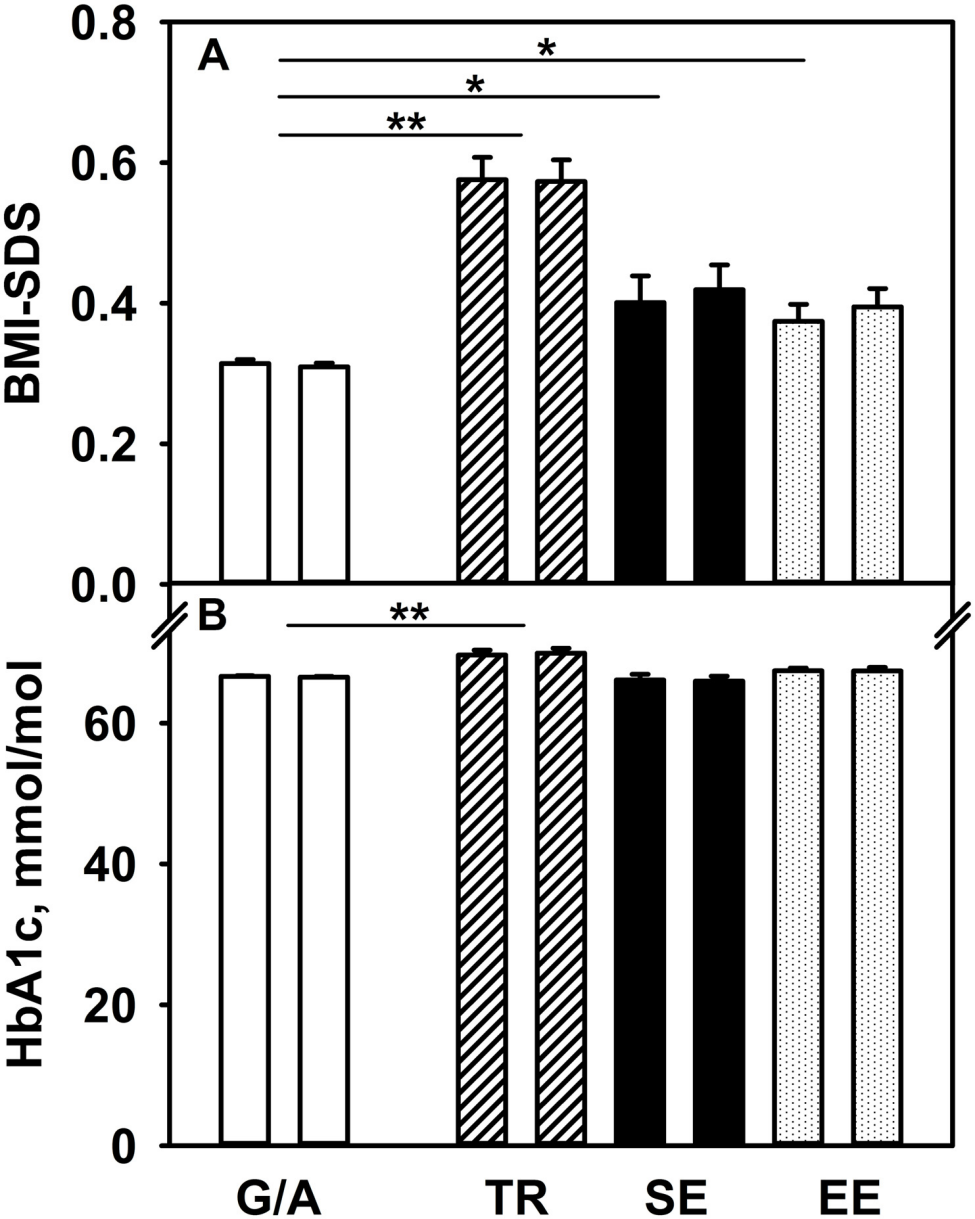
BMI-SDS (A) and glycemic control (B) in type-1-diabetes, depending on maternal (left bar) or paternal (right bar) country of birth. G/A Germany/Austria (white bar), TR Turkey (hashed bar), SE Southern Europe (black bar), EE Eastern Europe (dotted bar), *p<0.05, **p<0.001. Given are adjusted estimates (±SE) based on multivariable regression modeling. Adjustments were made for age, sex and duration of diabetes. To convert HbA1c in %: (mmol/mol-value ÷ 10.929) + 2.15.

BMI-SDS values of native and non-native children after adjustment for age, sex and duration of diabetes are summarized in Fig 2A. Compared to offspring of native mothers, patients with Turkish, Southern European, or Eastern European migrant background revealed a significantly higher BMI-SDS.

### Glycemic control

Fig 2B depicts glycemic control assessed by HbA1c for children of non-native mothers compared to offspring of native mothers after adjustment for age, sex and duration of diabetes. Descendants of Turkish mothers had a worse glycemic control compared to offspring of native mothers (Fig 2B). By contrast, in patients of mothers from Southern or Eastern Europe, no difference could be observed compared to offspring of native mothers (Fig 2B).

### Diabetes therapy

Data on insulin therapy are summarized in Table 1 and are adjusted for age, sex and duration of diabetes. The use of insulin pumps was less common and the number of injection time-points per day was lower in patients with migration background, irrespective of region of origin (Table 1). By contrast, insulin dosage per kilogram body weight and the daily number of SMBG measurements did not differ clinically relevant between groups (Table 1).

**Table 1:** Diabetes therapy and outcome in type-1-diabetes patients aged ≤20 years, depending on maternal country of birth.

		Maternal country of birth						
		Germany/Austria (reference)	Turkey	P-value	Southern Europe	P-value	Eastern Europe	P-value
**N**		25,107	736		517		1,283	
**Diabetes therapy**								
	**Insulin pumps, %**	37.9	26.8	<0.001	27.9	<0.001	32.6	<0.001
	**Injection time-points, per day**	6.48±0.01	5.87±0.08	<0.001	5.97±0.09	<0.001	6.28±0.06	0.002
		(n = 24,770)	(n = 730)		(n = 514)		(n = 1,276)	
	Insulin dosage, IU/kg*d	1.037±0.002	1.054±0.012	ns	1.033±0.014	ns	1.057±0.009	0.035
		(n = 24,770)	(n = 730)		(n = 514)		(n = 1,276)	
	**SMBG, per day**	5.88±0.01	5.72±0.08	ns	5.57±0.10	0.002	5.81±0.06	ns
		(n = 23,473)	(n = 717)		(n = 498)		(n = 1,244)	
**Acute diabetes complications**								
	**Severe hypoglycemia, per 100 pat.years**	16.13±0.02	15.51±0.14	<0.001	14.83±0.16	<0.001	22.09±0.13	<0.001
	**DKA, per 100 pat.years**	6.54±0.02	7.50±0.10	<0.001	7.13±0.11	<0.001	6.20±0.07	<0.001
**Chronic diabetes complications**								
	**Hypertension, %**	28.3	32.0	0.030	26.5	ns	32.0	0.005
		(n = 24,358)	(n = 729)		(n = 509)		(n = 1,252)	
	**Dyslipidemia, %**	39.9	44.1	ns	33.7	0.016	41.2	ns
		(n = 19,380)	(n = 560)		(n = 384)		(n = 1,019)	
	**Microalbuminuria, %**	12.0	14.1	ns	11.9	ns	11.3	ns
		(n = 18,243)	(n = 558)		(n = 370)		(n = 999)	
**Patient care**								
	**Hospitalization, per 100 pat.years**	48.5±0.4	57.2±2.7	<0.001	46.7±2.9	ns	52.0±1.9	ns
	**Duration of hospital stay, days per 100 pat.years**	441±1	433±7	ns	355±8	<0.001	427±5	0.019
	**Outpatient visits, per 100 pat.years**	388±1	416±7	<0.001	392±9	ns	396±6	ns
		(n = 22,124)	(n = 685)		(n = 471)		(n = 1,161)	

Based on multivariable regression, adjusted estimates (±SE) were calculated. Adjustments were made for age, sex, duration of diabetes. P-values are given for the comparison between people of native mothers (Germany/Austria) and offspring of mothers born in Turkey, Southern Europe or Eastern Europe. Abbreviations: *DKA* diabetic ketoacidosis, *ns* not significant, *pat*.*years* patient-years, *SMBG* self-monitoring of blood glucose.

### Acute diabetes-related complications

Table 1 depicts also information on hypoglycemia and diabetic ketoacidosis after adjustment for age, sex and duration of diabetes. Severe hypoglycemia was more common in patients of Eastern European descent compared to offspring of native mothers. By contrast, no clinically relevant difference in the occurrence of severe hypoglycemia was observed between offspring of Turkish or Southern European mothers and patients of native mothers. Diabetic ketoacidosis was more frequent in descendants of Turkish or Southern European mothers, whereas in offspring of Eastern European mothers the rate was slightly lower compared to children of native mothers.

### Chronic diabetes-related complications

Results of chronic diabetes complications after adjustment for age, sex and duration of diabetes are summarized in Table 1. Compared to offspring of native mothers, hypertension was more frequent in patients with Turkish or Eastern European background and tended to be less common in individuals of Southern European descent (Table 1). Dyslipidemia occurred less frequently in offspring of Southern European mothers and revealed a tendency towards a higher rate in offspring of Turkish mothers (Table 1). Between patients with Eastern European background and individuals of native mothers, the frequency of dyslipidemia was comparable (Table 1). Regarding the presence of microalbuminuria, no statistically significant difference could be found between groups (Table 1). However, a trend towards a higher frequency of microalbuminuria in children of Turkish mothers compared to offspring of native mothers could be observed (Table 1).

### Patient care

As presented in Table 1, the rate of hospitalization and the number of outpatient visits were higher in patients of Turkish descent, but comparable between offspring of Southern or Eastern European mothers and individuals of native mothers after adjustment for age, sex and duration of diabetes. Moreover, the duration of hospital stay was shortest in patients of Southern European descent, whereas no relevant difference could be observed between the other groups (Table 1).

### Impact of paternal country of birth

Table 2 summarizes the various combinations of maternal and paternal origin of birth. As indicated by bold-typed figures, the majority of the parents came from the same country.

**Table 2:** Cross-classified table for parental country of birth.

		Paternal country of birth					Total
Maternal country of birth, n/%		Germany/Austria	Turkey	Southern Europe	Eastern Europe	Unknown	
	**Germany/Austria**	24,631	89	161	78	148	25,107
		**98.1%**	0.4%	0.6%	0.3%	0.6%	100%
	**Turkey**	45	688	0	0	3	736
		6.1%	**93.5%**	0.0%	0.0%	0.4%	100%
	**Southern Europe**	84	1	420	3	9	517
		16.2%	0.2%	**81.2%**	0.6%	1.8%	100%
	**Eastern Europe**	188	10	18	1,041	26	1,283
		14.7%	0.8%	1.4%	**81.1%**	2.0%	100%
**Total, n/%**		24,948	788	599	1,122	186	27,643
		90.3%	2.8%	2.2%	4.0%	0.7%	100%

Data are given as absolute values (first line) and row percentages (second line). Bold-typed figures indicate that parents were from the same country. Of 186 patients, paternal origin of birth was unknown or not documented.

Repeating the analyses after categorizing individuals based on their paternal country of birth did not change our findings, except for dyslipidemia and insulin dosage. In contrast to the analysis of maternal migrant status, the non-significantly higher prevalence of dyslipidemia in individuals with Turkish background reached significance in the analysis of paternal migrant status (43.9% vs. 39.8%, p = 0.045). Furthermore, the significant difference in daily insulin dosage between children of Eastern European descent and native mothers lacked significance in the analysis of paternal migrant status (1.053±0.010 vs. 1.036±0.002 IU/kg*d, p = 0.099).

## Discussion

This large, multicenter study among T1D patients aged 20 years or younger revealed differences in diabetes therapy and outcome between patients with and without migration background, but also specific disparities among the three migrant groups studied. Besides a higher BMI-SDS and a lower frequency of insulin pump therapy in offspring with migrant background, differences in specific areas of diabetes therapy and outcome were observed depending on the region of origin. It seems that existing diabetes care structures do not adequately reach all patients with migrant background and should be modified in terms of diversity management.

The higher BMI-SDS in patients with migration background compared to offspring of non-migrants is in line with another German study indicating an increase in BMI among immigrants with longer duration of residence [32]. Several potential reasons are given in the introduction section. Moreover, cultural differences in the consumption of daily foods might be a further explanation. A representative German study reported for children with Turkish background a higher intake of soft drinks, white bread, chocolate and snacks compared to non-migrants [33]. Moreover, Russian migrants ate the highest amount of sausage and bacon [33]. The ideal body shape is assumed to be culture-sensitive [34] and thereby may also account for different BMI values between populations. Genetic differences between ethnicities may also play a role [15]. Recently, the ADA proposed the use of an Asian American-specific BMI cut-off for diabetes screening [35].

In our study, glycemic control was worse in patients of Turkish descent. Most research reported also a lower HbA1c in the respective native population than in minority groups [6,36]. A lower frequency of insulin injection time-points as observed by our study might be one explanation. For non-Scandinavian children, a lower number of bolus insulin injections and a slightly higher HbA1c compared to children of Scandinavian mothers had been reported [20]. When interpreting our results, physiological and biological differences between ethnic groups should also be considered (e.g. differences in red blood cell turnover, biological factors influencing non-enzymatic glycation or enzymatic deglycation of HbA1c independent of mean blood glucose [36]).

The results of our study suggest that diabetes outcome varies depending on type of migration background. For example, most diabetes complications were more prevalent in patients of Turkish mothers, whereas in people of Southern or Eastern European descent solely specific differences compared to offspring of native mothers were observed. A combination of different factors and a potentially inadequate adaption of existing care structures to the needs and risks of patients with migrant background are suspected to cause higher rates of diabetic ketoacidosis and hypoglycemia in some groups of migrants. Besides the previously mentioned reasons, the higher rate of hypoglycemia in patients with Eastern European background might be due to differences in eating and may be drinking patterns (e.g. higher intake of calories from fat, or higher consumption of alcohol with more irregular heavy drinking episodes [37]). As shown recently, there is no longer an inverse association between HbA1c and the risk of hypoglycemia [24].

Our findings confirm other studies suggesting a higher prevalence of hypertension and a more unfavorable lipid profile in people of Turkish origin [38,39]. The lower prevalence of dyslipidemia in individuals of Southern European descent is not surprising and might be related to differences in LDL receptor activity.

As indicated by a preliminary report focusing on the access to insulin pumps in patients with Turkish origin [22] and reported also for children of non-Scandinavian mothers [20], a less frequent use of insulin pumps was observed in offspring of non-native parents; with the lowest prevalence in patients with Turkish background followed by offspring of Southern European mothers and people of Eastern European descent. Unlike other health care services in Germany and Austria, the costs of insulin pump therapy are not automatically covered by the statutory health insurances. Each patient has to apply individually for reimbursement. Differences in approval of reimbursement between patients with and without migration background cannot be completely excluded, as poor integration and discrimination of migrants is still present [10]. Moreover, insulin pump therapy is more complex and requires a higher competence for self-management than conventional injection therapy [40]. All together may contribute to a lower use of insulin pumps in patients with migrant background. On the other hand, a lower education, a potentially lower rate of acceptance for pump therapy or new techniques and a possible lack of knowledge about health care offers might be further reasons.

Contrary to previous assumptions of a higher rate of hospitalization and a longer duration of stay in patients with migrant background [41], no major difference was observed between patients of native and non-native mothers. Although limitations in diabetes care for undocumented immigrant patients should be kept in mind, this is likely due to the highly standardized diabetes care in Germany and Austria. The only exception was the significantly higher rate of hospitalization and the higher number of outpatient visits in people of Turkish descent which may be related to the higher HbA1c and thus to more acute complications requiring hospital care (e.g. diabetic ketoacidosis).

Overall, the relatively small differences between patients with and without migration background found in our study compared to reports from other countries might be related to the statutory health insurance system existing in Germany and Austria that covers the majority of health care costs. Contrary to e.g. the US, access to health care services is not limited by differentials in insurance coverage.

Major strengths of the present study are the large number of patients included and the differentiation between regions of origin, as people with migrant background are not a homogenous group. One limitation is that migration status was defined by parental country of birth. Thus, patients of the “third generation” born in Germany or Austria to parents with a migration background were classified as offspring of native parents. Unfortunately, we could not analyze some of the individual-level variables e.g. parental support or patient satisfaction with insulin pumps that might explain the disparities between patients with and without migration background. Moreover, in several studies, differences attributed to migration background disappeared after adjustment for socio-economic status.

In conclusion, there is considerable heterogeneity in diabetes outcome among patients with migration background, but the differences between people with and without migrant background seem to be less pronounced in Germany and Austria than in other countries like the US or Canada. Nevertheless, in the future reasons contributing to differences in diabetes outcome should be investigated in order to tailor existing care structures to needs and risks of each individual patient irrespective of migrant background.
